# Effects of adverse social behaviour at the workplace on subsequent mental distress: a 3-year prospective study of the general working population in Norway

**DOI:** 10.1007/s00420-020-01581-y

**Published:** 2020-11-01

**Authors:** Tom Sterud, Therese N. Hanvold

**Affiliations:** National Institute of Occupational Health (STAMI), PO Box 5330 Majorstuen, N-0033 Oslo, Norway

**Keywords:** Psychosocial factors, Occupational exposure, Occupational stress, Workplace, Occupational health, Occupational groups

## Abstract

**Objective:**

We aimed to provide an integrated picture of the relationship between different facets of adverse social behaviour (ASB) at work and mental health problem.

**Methods:**

Data were provided from a longitudinal nationwide study of the general population in Norway. Eligible respondents were in paid work during a reference week in 2013, or temporarily absent from such work, and was interviewed at 3-year follow-up (*n* = 3654, response at baseline/follow-up = 53.1%/71.8%). We investigated the prospective associations of self-reported exposure to ASBs, including threats/acts of violence, bullying, sexual harassment and workplace conflicts, with mental distress (the Hopkins Symptoms Checklist) at follow-up, by means of multiple logistic regression.

**Results:**

In total, 6.6% (242 individuals) were classified with mental distress at follow-up. Work-related predictors were sexual harassment (OR = 1.64 07, 95% CI 1.03 − 2.61), bullying (OR = 2.07, 95% CI 1.19 − 3.60) and workplace conflicts (OR = 1.51, 95% CI 1.07 − 2.13). An elevated, but non-statistically significant association was observed for threats/acts of violence. No significant interactions were found between ASB and mental distress score at baseline. Overall there were few indications of substantial confounding related to age, sex, education level or occupation. After adjusting for these factors, the overall population attributable risk of mental distress attributable to any exposure to ASB was 11.3% (95%CI 0.6–22.3).

**Conclusions:**

We observed robust associations between exposure to three out of four types of ASB and risk of mental distress. Taken together, the results underscore that adverse social behaviour at the workplace may have a substantial impact on the level of mental distress in the general working population.

## Background

Mental disorders are among the leading causes of sickness absence and long-term work incapacity (Vigo et al. 2016). Over the past two decades, researchers have paid increasing attention to the relationship between different job characteristics and mental health problems, such as depression, anxiety and other stress-related conditions (Harvey et al. 2017). Less attention has been devoted to the likely mental health consequences of adverse social behaviour (ASB) at the workplace. This is somewhat surprising as the labour market shift towards the service industry implies that the relative significance of social stressors will increase with a surge of social interactions with patients, customers, clients and co-workers.

ASB in the workplace has been defined as any act of physical and verbal violence and intimidation at work, and includes acts of sexual harassment, bullying, threats/acts of violence or verbal abuse. According to the Sixth European Working Condition Survey, approximately 16% of workers in Europe reported exposure to one or more of these ASBs; the prevalence in Norway was 19% (Eurofound 2015). Judged by the number of systematic literature reviews, bullying appear as the most frequently studied aspect of ASB at the workplace. Based on a number of longitudinal studies (Bonde et al. 2016; Einarsen and Nielsen 2015; Figueiredo-Ferraz et al. 2015; Finne et al. 2011; Gullander et al. 2014; Kivimaki et al. 2003; Lange et al. 2020; Loerbroks et al. 2015; Nielsen et al. 2020; Rugulies et al. 2012), and systematic reviews of longitudinal studies (Theorell et al. 2015; Verkuil et al. 2015), it seem well documented that bullying at work increases the risk of mental distress. Longitudinal studies on the mental health consequences of sexual harassment, threats/acts of violence or workplace conflicts, however, are sparse (Hogh and Viitasara 2005; Lanctot and Guay 2014; McDonald 2012). Non-fatal violence and threats of violence have in a few longitudinal studies been reported to increase levels of fatigue (Hogh and Viitasara 2005), use of psychotropic drugs (Madsen et al. 2011), and risk of leaving the work-force (Friis et al. 2018). A meta-analyses of largely cross-sectional studies (McDonald 2012) and a handful of longitudinal studies (Hogh et al. 2016; Houle et al. 2011; Nielsen and Einarsen 2012; Taniguchi et al. 2016) support the inference of a possible association between sexual harassment and mental distress, whereas the literature on workplace conflicts as part of ASB is very limited (Theorell et al. 2015).

Assessing the impact of ASB on mental health remains a topic of great interest as the knowledge of how sexual harassment, threats/acts of violence and conflicts at work affect mental health are to a small extent elucidated in prospective studies of general working populations. Many studies on ASB may not be valid in the general working population because of specific sample characteristics. They are often based on non-probability samples, or focus on specific professions, with small samples (Hogh and Viitasara 2005; Lanctot and Guay 2014; McDonald 2012). Furthermore, different facets of ASB have been studied separately. We are not aware of studies that have examined these factors simultaneously, although they may not be mutually exclusive phenomena. Finally, most studies have focused on the relative risk alone, without considering the proportion of employees at risk. The importance of ASB for mental health problems in the working populations depends on both the relative risk estimates and the percentage of employees at risk, which is expressed as the population-attributable risk (PAR).

To address the research limitations outlined above, we aimed to comprehensively examine relationships between different facets of ASB and mental distress. We simultaneously tested the prospective association between sexual harassment, bullying, workplace conflicts and threats/ acts of violence and subsequent mental distress in a randomly sampled prospective cohort of the general working population in Norway.

## Methods

### Study design and study population

Data were provided from the ongoing nationwide Study of living conditions–work environment, conducted by Statistics Norway. Data were collected during the two periods; April 2013 to January 2014 and September 2016 to April 2017, and were collected by personal telephone interviews. Eligible respondents were community-living Norwegian residents aged 18–66 years. A gross sample of 20, 492 was randomly drawn from this population. Of these, 10, 875 persons responded in 2013 (response 53.1%). Due to a planned rotation in the panel selection, only two-thirds of these were invited to participate in 2016 (*n* = 7250). The non-response examination by Statistics Norway showed minor differences between non-responders and responders across the benchmarks of age, sex and geographic region, whereas non-response was higher among respondents with elementary education level (Statistics Norway 2014).

Among eligible respondents invited to participate at both surveys, 5573 were in paid work at baseline. Among these, 3999 (71.8%) responded at follow-up. We excluded respondents with missing values on the exposure variables (*n* = 266). In addition, we excluded respondents with missing values on occupation or education (*n* = 61) and mental distress (*n* = 18). Thus, the final sample consisted of *n* = 3654 individuals. We defined two (nested) samples to analyze the relationship between ASB and mental distress. Sample 1 includes all eligible respondents after excluding respondents with missing values (*n* = 3654). Sample 2 is nested within sample 1, and is limited to respondents who were in paid work at both baseline and follow-up (*n* = 3325). The rationale for doing similar analyzes of the time-lagged associations between exposure to ASB at baseline and mental distress at follow-up for sample 1 and sample 2 was to take into account a possible selection out of working life related to mental distress and exposure to ASB at baseline (i.e., a healthy worker effect in sample 2).

## Measurement

### Outcome

Mental distress during the last 2 weeks was measured by a short version of the Norwegian translation of the Hopkins Symptoms Checklist (HSCL-25) (Tambs and Moum 1993). This is a frequently used and validated screening tool designed to detect symptoms of anxiety and depression. A concordance rate of 86.7% was demonstrated between the assessment by the physician and the patient's own rating of distress on the HSCL-25 (Hesbacher et al. 1980). The HSCL-5 scores were estimated to correlate 0.92 with the total score from the original instrument (Tambs and Moum 1993). Sensitivity and specificity for HSCL-5 have been estimated at 82% and 96% (Strand et al. 2003). The HSCL-5 consists of five items: (i) feeling fearful, (ii) nervousness of shakiness inside, (iii) feeling hopeless about the future, (iv) feeling blue (v) worrying to much about things. Each with four response options, ranging from 1 = “not afflicted” to 4 = “very much afflicted”. The index was scored as the mean of the item scores. The suggested cut-off for the HSCL-5 of ≥ 2.0 was used to define cases with mental distress (Strand et al. 2003).

### Adverse social behaviour

Two main approaches for measuring ASB are common in the literature. The self-labelling method measures the respondents subjective perception of being a victim of ASB, usually by directly asking a single question of whether one has been exposed to ASB within a defined period of time (Hershcovis and Barling 2007). By contrast, the behavioural method measures ASB by means of a list of specified negative acts (Einarsen and Raknes 1997)). In this latter method, the victims’ exposure to ASB is determined by an operational criteria set by the researchers. A commonly used criteria are Leymann’s (1996) criterion; exposure to at least one negative act on a weekly basis over a period of 6 months.

In the present study, ASB was measured by the self-labelling method. We used a relatively broad definition in terms of frequency, as we classified all participants that did answer “yes, weekly or monthly” as exposed (see items below). This definition of bullying, has been shown to overlap with a single item measure of bullying (including a definition of bullying as repeated negative acts within a 6-month period) used in another validated questionnaire *(*Dallner 2000; Kleven and Normann 2009*)*. For sexual harassment or threats/acts of violence, a similar operationalization seem appropriate, as these acts may occur rarely or even at a single occasion.

The items covering bullying, sexual harassment, threats/acts of violence were originally developed by an Nordic expert group and have been asked in a survey of living conditions since 1989 (Orhede 1994), and have later been slightly modified. Sexual harassment was measured with a single item: “Do you sometimes: (Q1) receive unwanted sexual attention, comments, etc., at your workplace? (answer categories: yes, ≥ 1 a week; yes, ≥ 1 a month; no). Sexual harassment was dichotomized and those answering “yes” were the exposed.

Bullying was measured with two questions: “Do you sometimes: (Q1) get bothered or teased in an unpleasant way by your colleagues? (Q2) get bothered or teased in an unpleasant way by superiors?” (Answer categories: yes, ≥ 1 a week; yes, ≥ 1 a month; no). Bullying was dichotomized into exposed for the ones who answered “yes” on any of the two questions. Threats/acts of violence was measured with three items: “Over the past 12 months have you: (Q1) been the victim of violence at the workplace that caused visible marks or physical damage? (Q2) been the victim of violence at the workplace that did not cause visible marks? (Q3) been threatened at the workplace in such a way that you felt scared?” Answer categories were “yes” and “no”. The items were computed into one dichotomous variable (yes at any item = 1, no = 0). Workplace conflicts was measured with two questions: “Have you often, sometimes, rarely or never been involved in unpleasant conflicts with: (Q1) your superior or (Q2) your colleagues”. Workplace conflicts was dichotomized into exposed for the ones who answered “often” or “sometimes” on any of the two questions**.** Adverse social behaviour combined [ASB, combined] was defined as any exposure to ASB (yes at any item = 1, no = 0).

### Covariates

Sex, age and level of education were based on administrative registry data. Age and education were treated as continuous variables in the regression analyses, but was recoded into dummy variables for descriptive purposes (ISCO 0 − 2: Elementary level; ISCO 3 − 5: upper secondary education; ISCO 6: University/college 4 y; and ISCO 7 − 8: University/college 4 y +). Occupation was based on an open questionnaire and coded by Statistics Norway into professional title in accordance with the International Standard Classification of Occupations (ISCO-88). We used 1-digit codes and recoded into six occupational groups to obtain sufficiently large groups to present data (see Tables 1, 2, 3).

**Table 1:** Prevalence (%) of adverse social behaviour and mental distress by sex, age, education and occupation

Covariates / number of respondents		Adverse social behaviour (ASB) at baseline					Mental distress at follow-up
	*N*	Sexual harassment	Bullying	Threats/acts of violence	Workplace conflicts	ASB, combined	
Total	3654	4.9	3.0	7.1	13.4	22.1	6.6
Sex							
Men	1856	1.8	2.7	4.4	12.1	16.8	4.7
Women	1798	8.0	3.3	10.6	14.8	27.6	8.6
Chi square tests		75.2 (1)*	1.3 (1)^NS^	50.1 (1)*	6.1 (1)*	62.2 (1)*	22.8 (1)*
Age groups							
18–34	948	8.1	2.6	8.1	11.5	22.8	9.1
35–49	1488	4.6	2.7	7.3	15.5	23.6	5.8
50–66	1218	2.6	3.7	7.1	12.4	19.9	5.7
Chi square tests		35.0 (2)*	2.9 (2)^NS^	0.9 (2) ^NS^	9.7 (2)*	5.8 (2)*	12.4 (2)*
Education level							
Elementary level	431	8.6	5.1	7.4	12.1	23.7	8.3
Upper secondary education	1367	4.1	3.1	6.1	13.2	21.4	
University/college 4 y	1333	5.4	2.6	10.7	14.6	24.8	5.8
University/college 4 y +	523	2.5	2.1	2.7	12.0	16.3	4.7
Chi square tests		21.8 (3)*	8.6 (3)*	41.84 (3)*	3.2 (3)*	16.9 (3)*	5.3 (3) ^NS^
Occupational groups							
Legislators, senior officials, managers	420	1.4	2.4	5.7	16.4	21.4	5.0
Professionals	1335	5.0	2.5	8.4	13.8	22.5	6.2
Technicians and associate professionals	605	2.8	2.6	6.0	11.1	18.8	5.3
Clerks	217	4.1	3.7	3.2	11.1	15.2	9.2
Service-, shop-/market sales workers	601	11.3	4.0	14.1	14.1	32.4	10.1
Manual- and elementary occupations	476	2.3	3.8	1.7	13.0	16.2	5.3
Chi square tests		77.2 (5)*	5.1 (5)^NS^	73.2 (5)*	7.7 (5) ^NS^	56.9 (5)*	19.8 (5)*

*N* = number of respondents, *p ≤ 0.05, *NS* not statistically significant. *ASB* combined = any exposure to ASBs (yes at any of the four items)

**Table 2:** Multiple logistic regression: Mental distress at 3-year follow-up regressed on work-related adverse social behaviour (ASB) measured at baseline

	Sample 1 (*n*^§^ = 3654 / cases^£^ = 242)		Sample 2 (*n*^§^ = 3325 / cases^£ =^ 197)	
	OR	95%CI	OR	95%CI
Sexual harassment^A^	2.66	(1.75 − 4.05)	2.13	(1.30 − 3.47)
Sexual harassment^B^	1.71	(1.08 − 2.71)	1.42	(0.83 − 2.41)
Sexual harassment^C^	1.64	(1.03 − 2.61)	1.36	(0.80 − 2.33)
Bullying^A^	3.84	(2.34 − 6.28)	2.60	(1.41 − 4.79)
Bullying^B^	2.15	(1.24 − 3.71)	1.36	(0.69 − 2.69)
Bullying^C^	2.07	(1.19 − 3.60)	1.33	(0.67 − 2.65)
Threats/acts of violence ^A^	1.68	(1.12 − 2.52)	1.58	(1.01 − 2.50)
Threats/acts of violence ^B^	1.14	(0.74 − 1.77)	1.08	(0.66 − 1.76)
Threats/acts of violence ^C^	1.11	(0.71 − 1.73)	1.02	(0.62 − 1.68)
Workplace conflict^A^	1.98	(1.44 − 2.73)	1.67	(1.16 − 2.41)
Workplace conflict^B^	1.48	(1.05 − 2.08)	1.30	(0.88 − 1.91)
Workplace conflict^C^	1.51	(1.07 − 2.13)	1.32	(0.90 − 1.95)
ASB, combined	2.02	(1.53 − 2.67)	1.77	(1.30 − 2.42)
ASB, combined	1.45	(1.08 − 1.95)	1.26	(0.90 − 1.76)
ASB, combined	1.44	(1.06 − 1.94)	1.26	(0.90 − 1.76)

Sample 1 includes all eligible respondents after excluding respondents with missing values

Sample 2 is nested within sample 1 and limited to respondents in paid work at both time-points

ASB, combined = any exposure to ASBs (yes at any of the four items)

^§^ = total sample in the analyses

£ = number of cases with mental distress at follow-up

Reference value = not exposed (not shown)

A = adjustment for sex and age (continuous)

B + mental distress at baseline (continuous)

C + occupation and education level (continuous)

**Table 3:** Reversed associations: Adverse social behaviour at follow-up regressed on mental distress at baseline

	Sexual harassment		Bullying		Threats/acts of violence		Workplace conflicts		ASB, combined (*n*^§^ = 3322 / cases^£^ = 694)	
	(*n*^§^ = 3319 / cases^£^ = 119)		(*n*^§^ = 3286 / cases^£^ = 92)		(*n*^§^ = 3322 / cases^£^ 221)		(*n*^§^ = 3258 / cases^£ =^ 433)			
	OR	95%CI	OR	95%CI	OR	95%CI	OR	95%CI	OR	95%CI
Mental distress^A^	1.71	(0.92 − 3.18)	2.47	(1.29 − 4.74)	2.06	(1.29 − 3.26)	1.80	(1.23 − 2.65)	1.79	(1.29 − 2.49)
Mental distress^B^	1.10	(0.56 − 2.20)	1.84	(0.93 − 3.66)	1.48	(0.89 − 2.46)	1.48	(0.99 − 2.21)	1.31	(0.92 − 1.86)
Mental distress^C^	1.03	(0.51 − 2.09)	1.73	(0.87 − 3.47)	1.49	(0.89 − 2.49)	1.51	(1.01 − 2.26)	1.31	(0.92 − 1.86)

ASB, combined = any exposure to ASBs (yes at any of the four items)

^§^ = total sample in the analyses

£ = number of cases with exposure to adverse social behaviour at follow-up

Reference value = not exposed (not shown)

A = adjustment for sex and age (continuous)

B + exposure to the specific adverse social behaviour at baseline

C + occupation and education level (continuous)

### Statistics

The distribution of exposure to ASBs and mental distress by covariates (sex, age, education level and occupation) was described and tested for differences by means of Chi square tests. Furthermore, to address the issue of possible selection bias related to attrition, we tested if response at follow-up were dependent on exposure, outcome and confounder variables at baseline by means of Chi Square tests. We used a significance level of 0.05. The prospective associations between ASB and mental distress were calculated as odds ratios (ORs) with 95% confidence intervals (CIs). Adjustments for potential confounders were made by logistic regression analyses. Firstly, we computed three regression models in which mental distress at follow-up were regressed on ASBs measured at baseline. Model^A^ was adjusted for age and sex, whereas adjustments for mental distress at baseline were computed in model^B^. In the main model^C^, we further adjusted for occupation and educational level. Moreover, we did additional separate interaction analyses to test whether associations were modified by sex or mental distress at baseline (based on model^C^). To test for reverse associations, we regressed exposure to ASBs at T2 on mental distress at baseline. Finally, we calculated the population attributable risk percent (PAR%) with 95% CI (Table 4). Based on the method described by Natarajan et al. (Natarajan et al. 2007) PAR% was calculated using the formula Pd*((OR-1)/OR), where Pd is the proportion of cases (i.e., mental distress at the follow-up) exposed to a risk factor. The lower and upper limits of the 95% CI for PAR% were calculated from the general PAR% formula using the lower and upper limits of the 97.5% CI for Pd and OR. This constitutes the 95% Bonferroni CI for PAR%. Based on the assumptions that the model variables are statistically independent and there are no interactions in the logistic model, the summary attributable risk was calculated according to the formulae: 1 $-$(1 $-$ PAR_var1_)(1 $-$ PAR_var2_)(1 $-$ PAR_var3_) etc. (Bruzzi et al. 1985).

**Table 4:** Calculated population attributable risk (PAR%) based on ORs from Table 2 (Sample 1: *n* = 3654 / mental distress cases = 242)

	Model^A^	95%CI	Model^B^	95%CI	Model^C^	95%CI
	PAR%		PAR%		PAR%	
Sexual harassment	8.3	(3.3 − 13.9)	5.5	(0.1 − 11.8)	5.2	(−0.3 − 11.6)
Bullying	5.7	(1.9 − 10.2)	4.9	(0.6 − 9.9)	4.7	(0.5 − 9.8)
Threats/acts of violence	5.2	(0.4 − 11.0)	1.6	(−3.6 − 8.3)	1.3	(−4.0 − 8.1)
Workplace conflicts	11.3	(4.6 − 18.7)	7.3	(−0.1 − 15.6)	7.6	(0.3 − 15.9)
ASB, combined	18.8	(9.7 − 28.3)	11.5	(0.9 − 22.5)	11.3	(0.6 − 22.3)

*PAR%* Population attributable risk percentage

*ASB, combined* = any exposure to ASB (yes at any of the four facets of adverse social behaviour)

A = adjustment for sex and age (continuous)

B + exposure to the specific adverse social behaviour at baseline

C + occupation and education level (continuous)

## Results

In total, 3654 respondents were included in the statistical analyses. Among eligible respondents invited to participate at both surveys 1574 respondents (28.2 percent) did not respond at follow-up. Attrition was not significantly associated with mental distress at baseline, and no significant associations were observed for three out of four explanatory variables (analyses not shown). Sexual harassment was associated with higher response at follow-up (22% vs. 28% among non-exposed, *p* < 0.05). Non-response at follow-up was substantially higher among younger and older workers and among employees with lower education level (40% in elementary education vs. 20% in University/college 4 y + , *p* < 0.05). Employees in manual or elementary occupations and service workers were more likely of not responding than legislators, senior officials and managers (34–35% vs. 24%, *p* < 0.05). Furthermore, we tested whether missing values on at least one out of the four exposure variables was associated with mental distress at follow-up. In total 266 respondents had missing values, and their risk of mental distress at follow-up was close to unity (OR, _adjusted for age and sex, mental distress at baseline_ = 1.02 95%CI 0.56 − 1.85).

The prevalence of mental distress at follow-up was 6.6 percent. Mental distress was significantly associated with being a woman, younger age and lower level of education. The prevalence of ASBs was: sexual harassment (4.9%), bullying (3.0%), threats/acts of violence (7.1%), workplace conflicts (13.4%), and any exposure to ASB (22.1%). ASBs were more prevalent among women, younger workers, workers with lower level of education, and among service workers and shop and market sales workers compared to other occupational groups (Table 1).

Table 2 shows the results of multiple logistic analyses with baseline exposures as the predictors and mental distress at follow-up as the outcome. In the initial model^A^ (adjusted for age and sex), we observed time-lagged associations between all facets of ASB and mental distress, the associations ranged from OR = 1.68 (95%CI 1.12 − 2.52) for threats/acts of violence) to OR = 3.84 (95%CI 2.34 − 6.28) for bullying (sample 1). The time-lagged association between ASB and mental distress was consistently higher in sample 1 (all respondents with valid responses on ASB at baseline, and metal distress at both time-points) compared with sample 2 (nested within sample 1, but limited to respondents in paid work at both time-point). We emphasize the fully adjusted model^C^ for sample 1: sexual harassment and bullying was associated with a 1.64 (95%CI 1.03 − 2.61) and 2.07-fold (95%CI 1.19 − 3.60) increased odds of mental distress at T2, respectively. Workplace conflicts was associated with a 1.51-fold (95%CI 1.07 − 2.13) increase of odds. The estimated OR for threats/acts of violence was 1.11 (95%CI 0.71 − 1.71). ASB combined (i.e., exposure to any of the four facets of ASB) was associated with a 1.44-fold (95%CI 1.06 − 1.94) increased odds of mental distress.

In supplementary analyses (table not shown), we separately evaluated the risk for mental distress among men and women. A significant difference was detected for sexual harassment (women: OR = 1.37 95%CI 0.81 − 2.32 and men: OR = 3.83 95% CI 1.54 − 9.51, *p* < 0.01). No significant interaction with sex was observed for bullying, threats/acts of violence and workplace conflicts. We found no significant interactions between any exposure to ASB and mental distress score at baseline.

Table 3 shows that mental distress at baseline was significantly associated with all types of ASB at follow-up in the initial model^A^ (adjusted for age and sex). In the fully adjusted model^C^, a statistically significant reversed time-lagged associations was observed for workplace conflicts (OR = 1.51 95%CI 1.01 − 12.26). Similar, but non-significant, correlations were observed for bullying (OR = 1.73 95%CI 0.87 − 3.47) and threats/acts of violence (OR = 1.59 95%CI 0.89 − 2.49). No excess risk was observed for sexual harassment (OR = 1.03. 95%CI 0.51 − 2.06).

The estimated PAR% of mental distress due to ASB is shown in Table 4. In the crude model^A^ (adjusted for age and sex), the estimated PAR% ranged from 5.5% (95%CI 0.4 − 11.0) for threats/acts of violence to 11.3% (95%CI 9.7 − 28.3) for workplace conflicts. Based on the fully adjusted model^C^, the proportion of mental distress attributed to ASB, combined was PAR = 11.3% (95%CI 0.6–22.3). The individual PAR% estimates ranged from 1.3% (95%CI -4.0 − 8.1) for threats/acts of violence to 7.6% (95%CI 0.6 − 22.3) for workplace conflicts.

## Discussion

This study investigated the role of different facets of ASB at the workplace in the development of mental distress in a representative sample of the Norwegian workforce. Both exposure to ASB and risk of mental distress were related to sex, age, level of education and occupation. After adjusting for these variables, we observed a time-lagged association between exposure to three out of four types of ASB (sexual harassment, bullying and workplace conflicts) and risk of mental distress. An elevated, but non-statistically significant association was observed for threats/acts of violence. The associations between ASBs and mental distress was consistently stronger in the analyses which took into account a likely healthy worker effect during the follow-up period. We estimated that about eleven percent of cases with mental distress was attributed to any exposure to ASB.

The strengths of this study are that it is a large nationwide study using random sampling, it uses a prospective design with a comprehensive set of exposures measured, it includes a thorough control of confounding and it has a rather high response rate. Nevertheless, self-selection bias is probable when the non-responders differ from those who responds. The non-response examination by Statistics Norway showed minor differences between non-responders and responders (Statistics Norway 2014), and the attrition analyses in the present study showed that neither the outcome variable nor the exposure variables were significantly associated with lower probability of responding at follow-up. Furthermore, the risk of mental distress at follow-up among respondents with missing values on any of the exposure variables at baseline, was similar to the risk within the non-exposed group. Thus, overall there is limited reason to suspect that bias due to attrition or missing values has substantially influenced the observed results.

All data were collected by self-report and this may have influenced the results in different ways. Firstly, reporting bias (e.g., due to negative affectivity) influencing exposure and outcome measures may have inflated the estimates. However, adjusting for symptoms of mental distress at baseline should reduce the risk of reporting bias due to negative affectivity or other personality dimensions related to mental distress. Secondly, the self-report method used in this study could lead to an underestimation of both exposure and outcome. Compared to mailed questionnaires, telephone respondents may be apprehensive about the judgments of the interviewer, particularly when it comes to socially stigmatized actions such as bullying or mental health problems (Siemiatycki 1979). Moreover, the four questions regarding ASB were phrased differently, and only threats/acts of violence was specified with a fixed time frame (i.e., over the past 12 months). This may have contributed to systematic misclassification and lower statistical power to detect true associations. Finally, our exposure measures were developed by an Nordic expert group (Orhede 1994). The questions have later been slightly modified, and their construct validity have not been extensively tested. The items measuring bullying have been shown to overlap with a single item measure used in another validated questionnaire (Dallner 2000; Kleven and Normann 2009). The single item measure of sexual harassment covers one of three components constituting this construct (Fitzgerald et al. 1995), whereas single items have been reported to adequately measure threats/acts of violence and workplace conflicts in other studies (Bultmann et al. 2002; Hanson et al. 2009; Madsen et al. 2011). Statistically, the time-lagged associations between different facets of ASB were modelled separately. Although entering all factors into one model would adjust for confounding, it would also partial out explained variance that is shared between overlapping factors. From a theoretical point of view, it is reasonable to assume that different facets of ASB are intertwined. Thus, to investigate a comprehensive range of facets covering the ASB construct, we estimated the individual effects of each facet separately, and the combined effect of being exposed to any facet of ASB.

Our finding of a prospective association between exposure to sexual harassment and development of symptoms of mental distress contributes to the limited literature (Hogh et al. 2016; Houle et al. 2011; Nielsen and Einarsen 2012; Taniguchi et al. 2016). We found limited support for a reversed association between mental distress and tendency to report sexual harassment at follow-up, and the strength of the association was independent of the level of mental distress symptoms at baseline. Together, the present findings support the inference of an increased risk of experiencing clinically relevant symptoms of mental distress after experiencing incidents of sexual harassment at work. Somewhat surprising our results indicated a possibly stronger association between sexual harassment and mental distress in men than in women. Accordingly, Hogh et.al (Hogh et al. 2016) reported a statistically significant association between unwanted sexual attention from co-workers and increased risk of sick leave among men, but not among women in the Danish workforce. However, this is partly in contrast to a previous Norwegian study, which reported a significant association among women only (Nielsen and Einarsen 2012). Different definitions of sexual harassment is one reasonable explanation for the different results. In the present study, sexual harassment was defined as a subjective experience of receiving unwanted sexual attention, whereas in the other Norwegian study (Nielsen and Einarsen 2012), sexual harassment was measured according to the criterion-based method. Thus, a still unsolved question is whether men and women respond differently to acts of sexual harassment.

In line with previous research on bullying and mental distress, our results show that bullying is associated with subsequent mental distress (Einarsen and Nielsen 2015; Nielsen et al. 2020; Verkuil et al. 2015). Our study further contributes to the literature by demonstrating a stronger time-lagged association between bullying and mental distress in the analyses which took into account a likely healthy worker effect during the follow-up period. In line with this, previous research has established bullying as a risk factor for sick leave (Nielsen et al. 2016), and the notion that bullying increases the risk of leaving the workforce has some support in the literature (Nielsen et al. 2017; Sterud 2013).

A novel finding was the prospective associations between exposure to workplace conflicts and the risk of developing mental distress during follow-up among both men and women. Severe social conflicts as a risk factor for depression in the general working population, has some support in the literature (Stoetzer et al. 2009). Other studies have reported association between mental distress and conflicts with supervisors (Bultmann et al. 2002), and conflicts with fellow workers (Hanson et al. 2009) among men, but not among women (Bultmann et al. 2002; Hanson et al. 2009). In the present study, workplace conflicts was the most prevalent type of ASB, and represented the largest population attributable risk.

Threats/acts of violence was associated with mental distress in the crude model, but was no longer a significant predictor after adjusting for mental distress at baseline. This is partly in contrast to previous studies that have reported associations between exposure to work-related violence and prescription claims for anti‐depressants (Madsen et al. 2011; Friis et al. 2018; Dement et al. 2014). The rather long follow-up period combined with a single point measurement of mental distress at follow-up in the present study are possible explanations for the somewhat different results. Individuals exposed to physical workplace violence have been reported to have a higher number of visits to the general practitioner, and to be more likely to be prescribed antidepressants (Friis et al. 2018). Thus, one may speculate that adequate help-seeking behaviour and treatment may contribute to reduce the strength of the association between exposure and mental health problems over time, and that a shorter follow-up period could have shown different results. Moreover, we cannot rule out the possibility that the impact of workplace violence may differ across industries, and depend on both the severity and the frequency of the incidents. Larger sample size and more detailed exposure categorization will be needed to elucidate these issues in future studies.

Time-lagged (reversed) associations between mental distress at baseline and exposure to ASBs at follow-up was observed for three out of four facets of ASB. The prospective relationships were equally strong for bullying, threats/acts of violence and workplace conflicts (OR ≥ 1.5), but bullying and threats/acts of violence did not reach statistical significance in the adjusted models. The inference that mental distress predict bullying has some support in the literature (Verkuil et al. 2015). Our study adds to the literature by indicating that this inference may hold true also for other facets of ASB. Plausible explanations for these observations have been discussed in the literature. Anxious or distressed employees may in periods struggle with social interactions, and evoke behaviour in others that appear abusive of threatening (Zapf 1999). At the same time, individuals with high levels of mental distress may have a tendency to interpret other people’s statements and behaviour as hostile (Matthiesen and Einarsen 2001), and have less resources to cope with these situations in a constructive manner. Thus, the present study, support the notions about a vicious circle between ASB and mental distress, which has been set forward in previous research (Finne et al. 2011; Kivimaki et al. 2003).

## Conclusions

Bullying, sexual harassment and workplace conflicts were independent predictors of mental distress. An elevated, but non-statistically significant association was observed for threats/acts of violence. Collectively, the adverse social behaviours accounted for about 11 percent of the mental distress cases. Furthermore, mental distress predicted self-reported bullying, workplace conflicts and threats/acts of violence, indicating a possible vicious circle between adverse social behaviour and mental distress. Taken together, the results indicate that adverse social behaviour at the workplace are potentially important and modifiable risk factors for mental health. Thus, early identification and routines for dealing with adverse social behaviours in organizations are important.
